# Identification and Analysis of *PPO* Gene Family Members in *Paulownia fortunei*

**DOI:** 10.3390/plants13152033

**Published:** 2024-07-24

**Authors:** Zhenli Zhao, Fei Wang, Minjie Deng, Guoqiang Fan

**Affiliations:** 1 College of Forestry, Henan Agricultural University, Zhengzhou 450002, China; zhaozhl2006@126.com (Z.Z.); wf18237409239@163.com (F.W.); dengmj1980@126.com (M.D.); 2 Institute of Paulownia, Henan Agricultural University, Zhengzhou 450002, China

**Keywords:** *Paulownia fortunei*, polyphenol oxidase, gene expression, bioinformatics, stress

## Abstract

Polyphenol oxidase (PPO) is a common metalloproteinase in plants with important roles in plant responses to abiotic and biotic stresses. There is evidence that PPOs contribute to stress responses in *Paulownia fortunei*. In this study, PPO gene family members in *P. fortunei* were comprehensively identified and characterized using bioinformatics methods as well as analyses of phylogenetic relationships, gene and protein structure, codon usage bias, and gene expression in response to stress. The genome contained 10 PPO gene family members encoding 406–593 amino acids, with a G/C bias. Most were localized in chloroplasts. The motif structure was conserved among family members, and α-helices and random coils were the dominant elements in the secondary structure. The promoters contained many cis-acting elements, such as auxin, gibberellin, salicylic acid, abscisic acid, and photoresponsive elements. The formation of genes in this family was linked to evolutionary events, such as fragment replication. Real-time quantitative PCR results showed that *PfPPO7*, *PfPPO10*, *PfPPO1*, *PfPPO2*, *PfPPO3*, *PfPPO4*, *PfPPO5*, and *PfPPO8* may be key genes in drought stress resistance. *PfPPO1*, *PfPPO3*, *PfPPO4*, and *PfPPO10* were resistant stress-sensitive genes. These results provide a reliable basis for fully understanding the potential functions of these genes and the selection of resistance breeding.

## 1. Introduction

Polyphenol oxidase (PPO) is a kind of metalloproteinase combined with copper ions that is commonly found in plants, bacteria, and fungi [1]. The PPO protein is generally composed of an N-terminal catalytic domain, PPO1_DWL domain, and PPO1_KFDV domain [2,3]. PPO1_DWL is a conserved sequence motif with a length of about 50 amino acids; this domain is critical for polyphenol oxidase activity [4]. The tyrosinase domain is about 210 amino acids, including CuA and CuB binding regions [5]. The PPO gene family includes seven PPO genes in tomato [6], three PPO genes in chestnut [7], and nine PPO genes of potato [8].

In plants, PPO catalyzes the oxidation of monophenol and resorcinol compounds to produce obenzoquinone, which is accompanied by oxygen reduction to water to produce brown-black pigments and protein complexes [9]. As a reactive oxygen species scavenger, PPO activity is an important indicator of plant stress resistance [10]. For example, cold injury can increase the expression of *PINPPO1* and *PINPPO2* in pineapple tissues [11]. Drought significantly induces *PtrPPO 9/13* and inhibits *PtrPPO 11/14/15* in poplar [12]. In strawberry, *FaPPO1* and *FaPPO2* gene expression levels are regulated by low temperature, abiotic stress (including NaCl and H_2_O_2_), and biotic stress (e.g., powdery mildew and gray mold) [13].

PPO contributes to plant defense responses via the production of quinones [14] or by catalyzing lignin to form protective shielding [15]. In tomato (*PPOF*), pineapple *(PINPPO1*), and soybean (*GmaPPO12*), PPOs increase resistance to fungi [12,16,17]. PPO gene family members in tobacco and Salvia miltiorrhiza are highly responsive to methyl jasmonate (MeJA) [18,19]. Antisense PPO gene expression in tomato can negatively regulate members of the gene family and produce hypersensitivity to pathogens [20]. The successful transfer of exogenous PPO into apple and potato explants significantly improves disease resistance [21]. *PtrPPO13* expression is significantly up-regulated when poplar is injured or infected with pathogens [22].

*Paulownia fortunei* is native to China and plays important roles in wood production as well as farmland and environmental protection [23]. Owing to its economic and ecological value, it has been introduced into many countries [24,25]. The species is susceptible to phytoplasma infection during its growth and development, resulting in a large number of clumps of indefinite buds, thereby reducing the yield and quality of Paulownia wood substantially. In view of the large differences in PPO genes between Paulownia and other species found in our previous studies of stress-related genes, we hypothesized that PPO activity contributes to the response to stress [26]. Therefore, in this study, PPO gene family members were identified from the whole genome sequence of *P. fortunei*, followed by analyses of sequence characteristics, phylogenetic relationships, and expression patterns under stress. Genes related to abiotic stress were screened to provide a theoretical basis for revealing the functions of PPO family members in *P. fortunei.*

## 2. Results

### 2.1. Identification of PPO Gene Family Members in P. fortunei and Analysis of Physicochemical Properties

**Figure 1:**
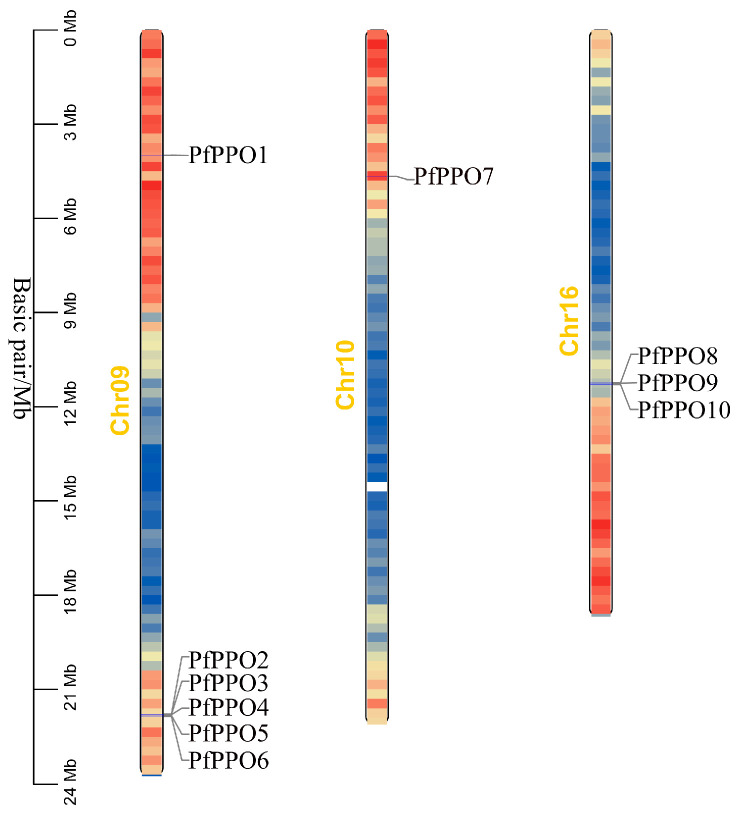
Distribution of *P. fortunei* PPO gene family on different chromosomes. Note: Color indicates the expression size of the gene, larger expression is darker, red is high expression and blue is low expression.

Ten PPO genes were identified using Pfam, HMMER, BLAST, and NCBI-CDD; they were named *PfPPO1* to *PfPPO10* according to their positions on the genome (Figure 1).

The physical and chemical properties of *PfPPO* proteins were analyzed using ProtParam. As shown in Table 1, among the 10 *PfPPOs*, *PfPPO10* was the shortest (406 amino acids) and *PfPPO8* was the longest (593 amino acids). The molecular weights of *PfPPOs* ranged from 44.959 to 66.677kDa. The isoelectric points (pI) ranged from 5.68 to 9.15, and the pI values for seven *PfPPOs* were lower than 7.00, indicating that most family members were acidic. The hydrophilicity of *PfPPOs* ranged from −0.533 to −0.357, indicating that all proteins in this family are hydrophilic.

The proteins encoded by the 10 *PfPPOs* were not predicted to contain signal peptides and belonged to non-secreted proteins, which do not enter the endoplasmic reticulum and Golgi secretion pathway after synthesis and are released directly into the cytoplasm. With the exception of *PfPPO5* and *PfPPO7*, the other eight proteins contained chloroplast transport peptides. With the exception of *PfPPO9*, the other nine family members are likely to have a transmembrane region. According to the above results and a subcellular localization analysis, *PfPPO5* was localized in the peroxisome, *PfPPO9* was located in the cytoplasm, and the other eight proteins were located in chloroplasts. This indicates that *PfPPOs* mainly function in chloroplasts, consistent with the results of subcellular localization analyses of the family in other species [27].

### 2.2. Analyses of Conserved Motifs and Gene Structure

**Figure 2:**
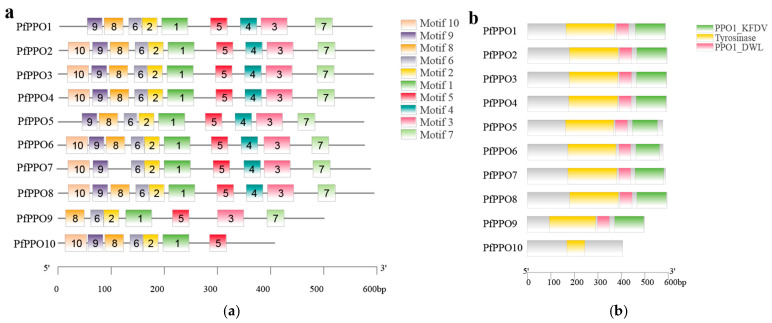
(**a**) Analyzing the conserved motifs of *PfPPO* gene family members; (**b**) the conservative domain analysis.

To study the structural characteristics of PPO gene family members of *P. fortunei*, the domain, conserved motif, and secondary structure were analyzed. Motif analyses (Figure 2a) revealed that all family members had motif1, motif2, motif5, and motif6, indicating that these four motifs were relatively conserved in the evolution of the PPO gene family. The high frequency of the motif structure motif10, motif9, motif8, motif6, motif2, motif1, motif5, motif4, motif3, and motif7 may be related to gene functions. Among the 10 motifs, only motif1, motif2, motif5, and motif6 were detected in all *PfPPOs*, indicating that these four motifs were the most highly conserved.

By analyzing the CDD of NCBI, the conserved domains of the amino acid sequences of *PfPPOs* were obtained (Figure 2b). All proteins contained the PPO1_DWL (PF12142.11) domain, except *PfPPO10*. All except *PfPPO9* and *PfPPO10* contained the tyrosinase (PF00264.23) domain, and all except *PfPPO6* and *PfPPO10* contained the PPO1_KFDV (PF12143.11) domain.

**Figure 3:**
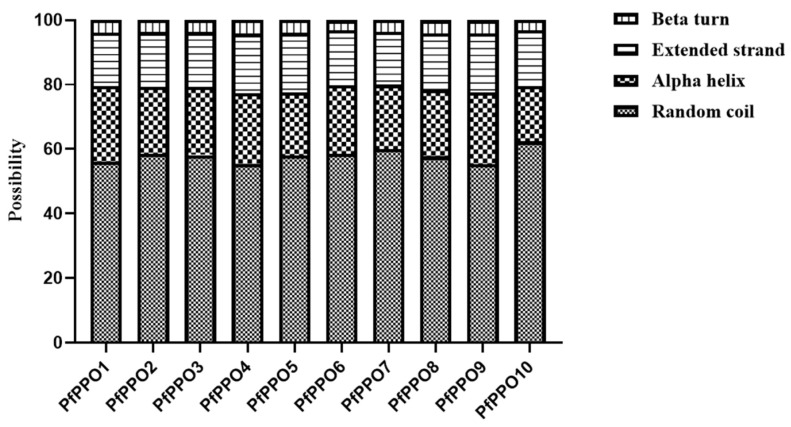
Stacking bar chart of percentage of secondary structure content of *PfPPOs*.

The secondary structure provides insight into protein function. The SOPMA online database was used to predict the secondary structures of 10 *PfPPO* proteins. The secondary structures of *PfPPOs* mainly included α-helices (17.24–23.51%) and random coils (55.31–62.32%), with relatively small frequencies of extended chains and β-turns (Figure 3). The α-helix in *PfPPOs* may contain the active site, and the β-fold contains sites for interactions with the catalytic substrate or inactivation [28].

### 2.3. Analysis of PPO Gene Codon Preference in P. fortunei

CUSP and CodonW1.4.4 were used to analyze the base content in the PPO gene coding region of *P. fortunei*. As shown in Table 2, the average GC content of three codon positions in PfPPOs’ gene was 52.01%. The average values for GC1, GC2, and GC3 were 50.37%, 48.65%, and 54.80%, respectively, revealing differences between GC1 and GC2 (with values similar to the average GC value across all sites) and GC3. The codon ENC in A/T-ending codons is a new index of bias [29]. The G and C contents of *PfPPO6*, *PfPPO8*, and *PfPPO10* were higher than the A and U contents, while the opposite pattern was observed for the other six genes. The GC and GC3s values for *PfPPO1*, *PfPPO7*, and *PfPPO9* were all lower than 50%, indicating a general preference for codons ending in G/C.

CodonW1.4.4 was used to calculate the relative codon usage in the PPO gene family. As shown in Table 3, the most commonly encoded amino acid was leucine (Leu) (463 codons or 8.25% of the total codons), followed by proline (Pro) (424 codons or 7.56% of the total codons). However, the fewest codons encoded tryptophane (Trp) (i.e., 85 codons). In the PPO gene family, 31 codons had RSCU values greater than 1, indicating a high frequency, and most of these ended in G and C. The RSCU values of UUG, GUG, UGA, and AGG were greater than 1.5, indicating a strong preference. Among these, the RSCU values of UUG and UGA were 1.80, and their codon usage frequency was the highest, indicating the strongest preference for these three codons.

### 2.4. Gene Structure and Promoter Element Prediction of PPO Gene Family Members of P. fortunei

**Figure 4:**
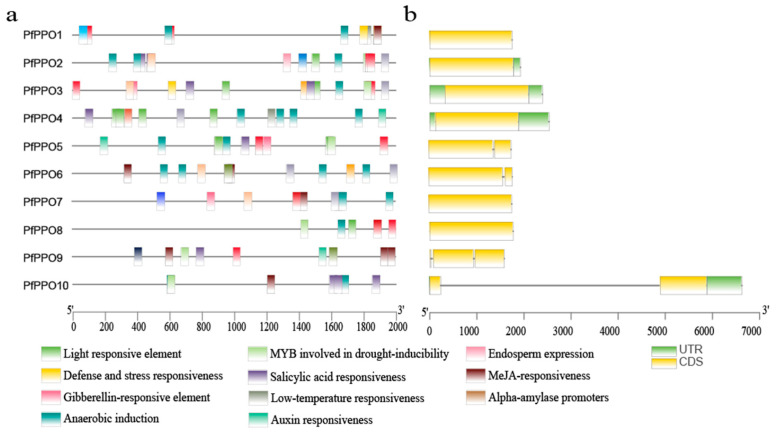
(**a**) Schematic diagram of cis-acting elements contained in *PfPPO* gene promoter region; (**b**) gene structure analysis.

The gene structures of 10 *PfPPOs* were analyzed using GSDS software (http://gsds.cbi.pku.edu.cn/ (accessed on 28 September 2022)). Six PfPPO genes had no introns (Figure 4b). The remaining four contained one or two introns; however, these were much shorter than the exons. Six genes had no non-coding region, two genes had non-coding regions at both the 5′ and 3′ ends, and two genes had non-coding regions only at the 3′ end. These results indicated that the PPO genes of *P. fortunei* show substantial diversification.

**Figure 5:**
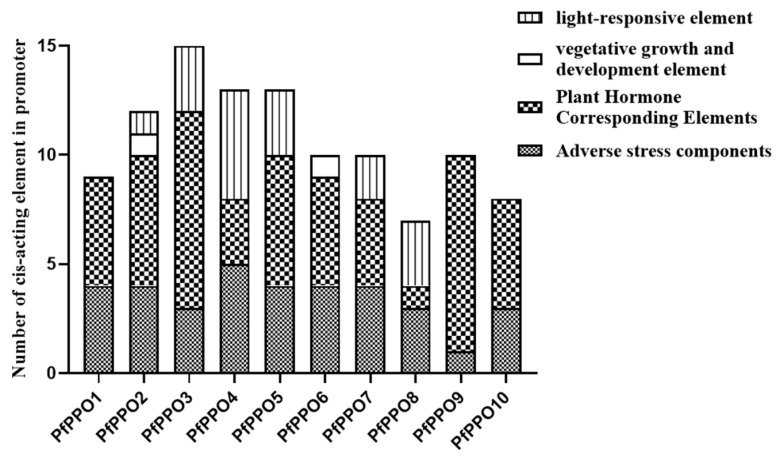
Cis-acting regulatory elements in *PfPPO* promoters.

The sequences 2000 bp upstream of 10 *PfPPOs* were analyzed using PlantCARE. We found that photoresponsive elements and hormone elements were widely present in *P. fortunei* PPO family members. Additionally, there were many cis-acting elements that respond to hormones (abscisic acid, salicylic acid, gibberellin, auxin, and photoallergens) and abiotic stress (low temperature) as well as those related to anaerobic induction, defense and stress responses, metabolic regulation, meristem expression, endosperm expression, heterologous regulation and circadian control, protein binding sites, and α-amylase promoters (Figure 4a and Figure 5). Various cis-acting elements were unevenly distributed across *PfPPOs*; for example, auxin-related elements were only present in the promoters of two genes, while salicylic acid-related elements were present in the promoters of six genes.

### 2.5. Collinearity and Phylogenetic Analyses of the PPO Family

**Figure 6:**
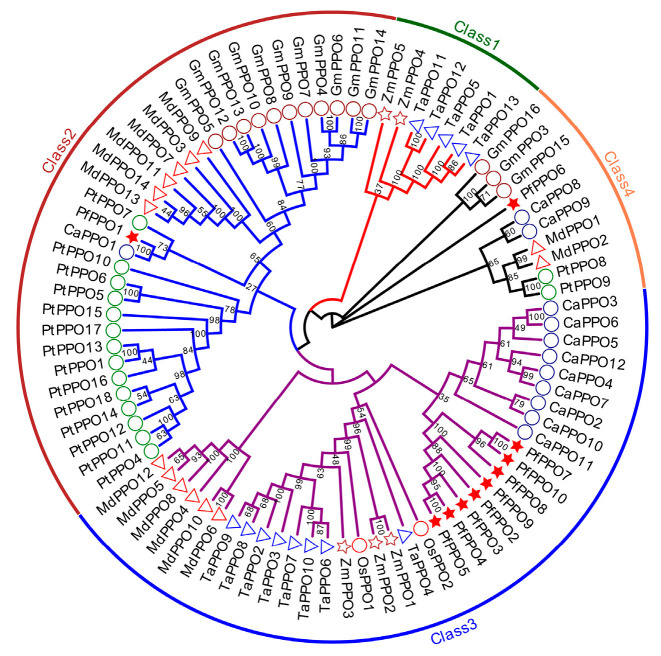
Phylogenetic tree of *PfPPO* family. Note—★: represents *P. fortunei*; **○**: represents *P. trichocarpa*; ○: represents *O. sativa*; ○: represents *C. annuum*; ○: represents *G. max*; **☆**: represents *Z. mays*; △: represents *T. aestivum*; △: represents *M. domestica*.

Phylogenetic trees were constructed based on amino acid sequences of 10 family members of *P. fortunei* as well as *Populus trichocarpa*, *Oryza sativa* L., *Capsicum annuum*, *Glycine max*, *Triticum aestivum*, *Zea mays*, and *Malus domestica* (Figure 6). *PfPPO1* and *CaPPO1* were closely related, and *PfPPO6* was closely related to *CaPPO8* and *CaPPO9*. The PPO gene family in *P. fortunei* was closely related to the PPO gene family in pepper.

**Figure 7:**
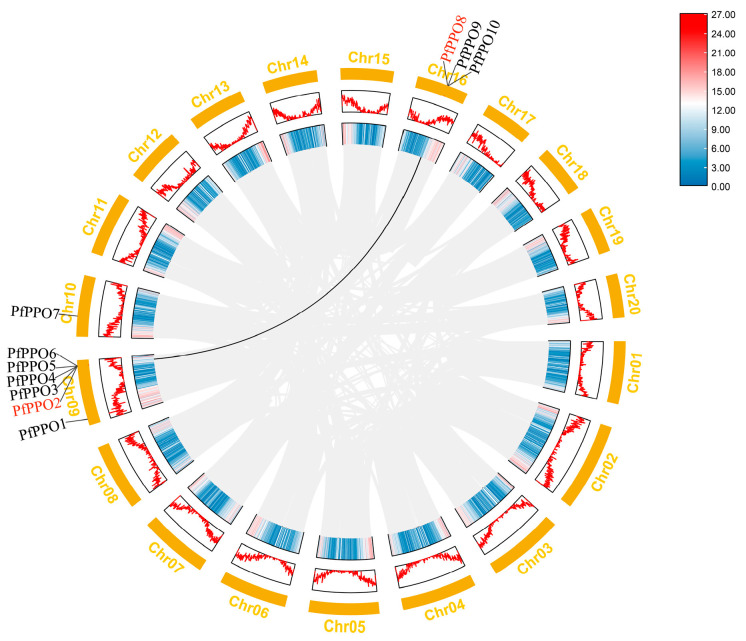
The collinearity analysis of *PfPPOs*. Note: Black lines indicate segmental duplicated gene pairs, and gray lines connect syntenic blocks in the genome. The inner circle represents the gene density of the chromosome. The exterior circle represents the amount of expression of the gene.

A collinearity analysis showed that *PfPPO2/3/4/5/6* on chromosome 9 and *PfPPO8/9/10* on chromosome 16 formed two tandem repeat regions. The high sequence similarity between the duplicate gene pairs suggested that they are involved in regulating similar biological processes. We also found that two genes (*PfPPO2* and *PfPPO8*) were fragmented repeats. These results suggest that *PfPPOs* expanded by gene duplication, and tandem duplication is the main driving force for the formation of the PPO gene family in *P. fortunei* (Figure 7).

### 2.6. Tissue Expression Analysis of PPO Gene Family Members of P. fortunei

**Figure 8:**
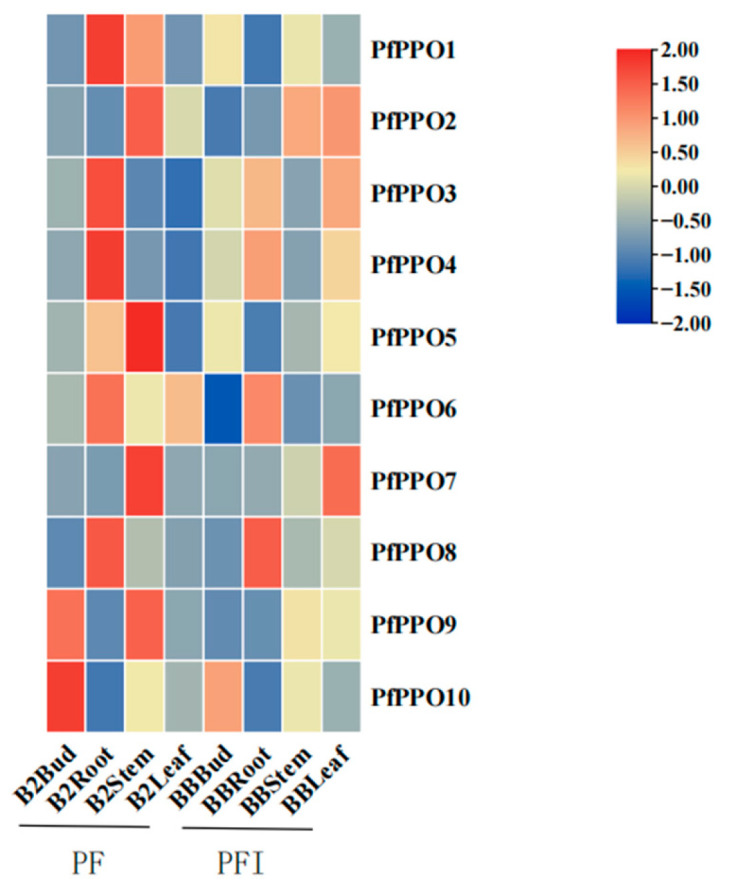
Relative expression of *PfPPO* genes in different parts of *P. fortune* in healthy (PF)and phytoplasma-infected (PFI)seedlings.

The genes showed differences in expression among tissues (Figure 8). The expression levels of *PfPPO1*, *PfPPO3*, *PfPPO4*, *PfPPO6*, and *PfPPO8* were highest in the root; *PfPPO2*, *PfPPO5, PfPPO7*, and *PfPPO9* were highest in the stem; and *PfPPO10* was highest in the bud.In addition, the expression levels of different genes varied within the same tissue. In the root, the expression of *PfPPO4* was the highest, and *PfPPO7* and *PfPPO9* were nearly undetectable. In the bud, the expression of *PfPPO10* was the highest, and *PfPPO7* was nearly undetectable. The expression of *PfPPO2* was the highest in both stems and leaves.

### 2.7. Effects of Witches’ Broom on Gene Expression in P. fortunei

To understand the roles of PPO gene family members in the pathogenesis of Paulownia witches’ broom, qRT-PCR was used to evaluate expression levels in different organs of infected seedlings (Figure 8). Compared with expression levels in healthy seedlings, the expression levels of *PfPPO1* and *PfPPO3* in the root were significantly lower and the expression levels of *PfPPO4* and *PfPPO6* were slightly lower in infected plants, while the expression levels of other genes did not differ significantly between infected and healthy plants. In the stem, all members showed variable decreases in expression. In leaves, *PfPPO3*, *PfPPO4*, and *PfPPO7* were up-regulated and showed the greatest expression changes. *PfPPO10* showed the most significant expression change in the bud. Overall, *PfPPO1*, *PfPPO3*, *PfPPO4*, and *PfPPO10* are the four PPO gene family members that respond positively to arbusardosis.

### 2.8. Responses of PPO Genes to Drought Stress in P. fortunei

**Figure 9:**
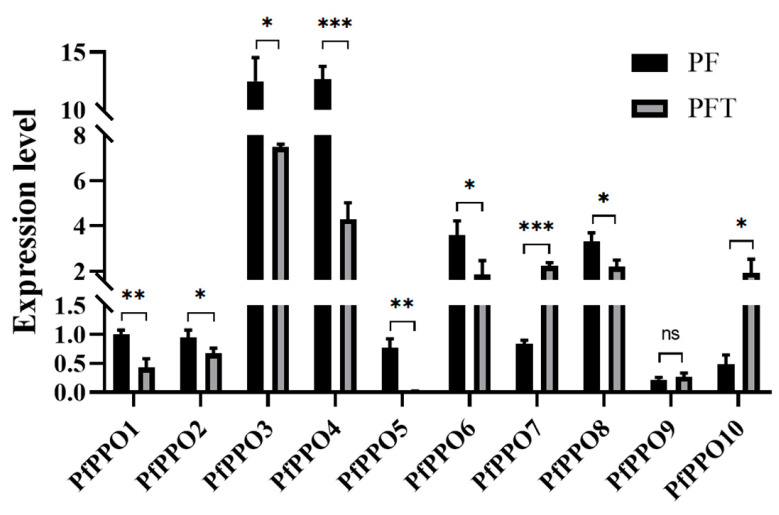
Relative expression of differential genes between control group and drought-treated seedlingsin *P. fortunei.* Note: * represents significant indigenous difference between PF and PFT (* *p* < 0.05, ** *p* < 0.01, and *** *p* < 0.001).

PPO as an antioxidant enzyme contributes to plant responses to abiotic stress [30]. Therefore, we evaluated the expression changes in PPO family members in *P. fortunei* before and after drought stress. As shown in Figure 9, except for *PfPPO9*, significant changes were found in the expression of the other nine family members, including highly significant changes in levels of *PfPPO1*, *PfPPO4*, *PfPPO5*, and *PfPPO7* under drought stress. In terms of trends, *PfPPO7*, *PfPPO9*, and *PfPPO10* expressions were significantly higher and the other seven expressions decreased compared to the untreated samples. Three genes (*PfPPO1*, *PfPPO3*, and *PfPPO4*) that were up-regulated in the pathogenesis of witches’ broom were down-regulated in response to drought stress, suggesting that members of the PPO family have different functions in response to biotic and abiotic stresses.

## 3. Discussion

PPOs are copper-containing enzymes that function as antioxidants and contribute to plant defense. In this study, 10 *PfPPO* genes were identified in *P. fortunei* using bioinformatics methods, with evidence for duplication events in the evolution of the gene family. Most encoded hydrophilic weakly acidic proteins, and the secondary structures, were dominated by α-helices and irregular coils. Localization was mainly in chloroplasts. All family members contained highly conserved motifs and domains. The hormone response elements showed uneven distributions in PPO gene family promoter sequences. These family members showed different responses to phytoplasma infection and drought, suggesting both conserved and divergent functions [31].

Recent studies have identified 18, 7, 12, 9, and 13 PPO genes in poplar, tomato, tobacco, potato, and wheat, respectively [6,12,20,27,32,33], compared with 10 PPO gene family members in the genome of *P. fortunei*. Other antioxidant enzyme gene families have fewer family members. For example, there are two CAT family genes in barley [34]; three CAT genes in Arabidopsis, rice, and maize [35,36,37]; four CAT genes in cucumber and soybean [38,39]; and seven in cotton and tobacco [40,41]. There are 15, 6, 9, and 18 SOD genes in pepper, corn, Arabidopsis, and tobacco, respectively [42,43,44,45], and 15 SOD genes in potato, tomato, and cucumber [46,47,48]. There are also some larger transcription factor families in plants. For example, the bZIP gene family has 89, 78, and 54 members in *P. fortunei*, Arabidopsis, and cayenne pepper [49,50,51]. The MYB gene family has 138, 152, 192, and 463 genes in *P. fortunei*, poplar, Arabidopsis, and soybean, respectively [52,53,54,55]. The number of gene family members is commensurate with its evolution, structure, and function, with relatively high numbers of genes in functionally diverse gene families.

Plant PPOs could be divided into four classes. *PfPPOs* were assigned to the second, third, and fourth categories, among which *PfPPO1* belonged to the second class. Most *PfPPOs* (*PfPPO2* to *PfPPO5* and *PfPPO7* to *PfPPO10*) are in the third class, and *PfPPO6* is in the fourth class. PPOs of the dicotyledonous plants Capsicum and apple are distributed in these three classes; PPOs of monocotyledonous wheat and maize belong to the first and second classes, and those of monocotyledonous rice belong to the third class. PPO families of each species are more likely to be closely related, indicating that PPO gene families show relatively low conservation. However, we found that *PfPPO1* and *CaPPO1* were closely related, and *PfPPO6* was closely related to *CaPPO8* and *CaPPO9*. Broadly, the PPO gene family in *P. fortunei* was most closely related to the PPO gene family in pepper.

Under drought stress, PPO gene activity in *Populus trichocarpa* is induced or inhibited to some extent [12]. A transcriptome analysis of *P. fortunei* showed that the expression levels of *PfPPO7* and *PfPPO10* were significantly higher, while the expression levels of *PfPPO1*, *PfPPO2*, *PfPPO3*, *PfPPO4*, *PfPPO5*, and *PfPPO8* were significantly lower after drought stress than without drought stress, similar to results in poplar. In *Malus domestica* sprouts, PPO gene activity is altered by infection with pathogens [21]. In *P. fortunei*, *PfPPO1*, *PfPPO3*, *PfPPO4*, and *PfPPO10* showed expression changes in response to witches’ broom, suggesting that these four genes contribute to disease resistance. At the same time, three genes (*PfPPO1*, *PfPPO3*, and *PfPPO4*) were up-regulated in witches’ broom and down-regulated in response to drought stress, suggesting that genes in the family may have different functions in response to biotic and abiotic stresses.

## 4. Materials and Methods

### 4.1. Plant Materials

We selected for the Henan Agricultural University tung biotechnology laboratory diploid healthy *P. fortunei* seedlings (PF), PaWB-infected *P. fortune* seedlings (PFI), and drought-treated *P. fortunei* seedlings (PFT; soil relative water content for field water at more than 80% for the control group, the soil relative water content for field water at about 50% for drought stress treatment group; drought stress after 15 d; collect different treatment groups of root, stem, leaf, and top bud). Among them, the culture method of healthy seedlings and PaWB-infected seedlings followed Cao et al.’s method [56]. The drought-treated seedlings were watered regularly every day, the control group supplied 100 mL normally, and the drought stress-treated group supplied 50 mL water. The culture temperature was (25 ± 2) °C, the light intensity was 130 μmol m^−2^s^−1^, and the light cycle was 16 h/8 h (light/dark). When histculture seedlings grew to 30 d, the roots, stems, leaves, and top buds of different groups were taken. After sampling, all samples were immediately flash-frozen with liquid nitrogen and stored in an ultra-low-temperature refrigerator at −80 °C.

### 4.2. P. fortunei PPO Gene Family Identification and Physicochemical Analyses

Genomic data for *P. fortunei* were downloaded from NCBI (https://www.ncbi.nlm.nih.gov/ (accessed on 15 September 2022)) to identify the *PfPPO* gene family. The hidden Markov model files for domains PPO-1(PF00264.23), PPO-2 (PF12142.11), and PPO-3 (PF12143.11) were downloaded from the Pfam database as seed sequences. HMMER3.0 was used to search all protein sequence files of *P. fortunei* (E-value < 0.001) [57], and candidate members of the *PfPPO* gene family were obtained. Then, the Ensembl Plants website (https://plants.ensembl.org/index.html (accessed on 15 July 2022)) was used to obtain PPO amino acid sequences for *Populus trichocarpa*, *Oryza sativa*, and *Nicotiana tabacum*. The query sequences were compared with the amino acid sequence file for *P. fortunei* (E-value < 0.001), and candidate members of the *PfPPO* gene family were obtained. After the two results were merged to remove redundant sequences, the NCBI-CDD online tool (https://www.ncbi.nlm.nih.gov/cdd/?term= (accessed on 25 June 2022)) was used for PPO domain validation, and members of the *PfPPO* gene family were finally identified. They were named according to their chromosomal sequence. TBtools (V 1.09868) [58] was used to draw the distribution of PPO gene family members on chromosomes, using default parameters.

EXPASY (httos://www.expasy.org/protparam/ (accessed on 27 June 2022)) was used to predict physical and chemical properties. CELLO (CELLO v.2.5) and WoLF PSORT (https://www.genscript.com/wolf-psort.html (accessed on 17 August 2022)) were used to predict the subcellular localization of members of the *PfPPO* gene family.

### 4.3. Analysis of Signal Peptides and Transmembrane Domains of PPO Family Members of P. fortunei

Chloroplast transport peptides from 10 *PfPPOs* were predicted using the Ipsort Server (https://ipsort.hgc.jp/ (accessed on 17 June 2022)).

SignalP 6.0 Server (https://services.healthtech.dtu.dk/services/SignalP-4.1/ (accessed on 17 August 2022)) was used to predict amino acid sequences. Signal peptide analyses were performed according to the default neural network method and hidden Markov models.

TMHMM Server v. 2.0 (https://services.healthtech.dtu.dk/services/TMHMM-2.0/ (accessed on 24 September 2022)) was used to predict membrane proteins and related domains.

### 4.4. Prediction of PPO Gene Family Domains

MEME online software (http://meme-suite.org/tools/meme (accessed on 25 September 2022)) was used for a motif analysis of the amino acid sequence of PPO gene family members of *P. fortunei*. The motif number was set to 10. Then, TBtools was used for a visual analysis of motifs.

Conserved domains were analyzed using CDD (Conserved Domain Database) and the results were visualized using TBtools.

SOPMA (https://npsa-prabi.ibcp.fr/cgi-bin/npsa_automat.pl?page=npsa_sopma.html (accessed on 25 September 2022)) was used to predict the secondary structure of PPO proteins in *P. fortunei*.

### 4.5. Codon Usage Characteristics

EMBOSS CUSP (http://emboss.sourceforge.net/ (accessed on 26 September 2022)) was used to calculate GC contents at the first (GC1), second (GC2), and third bases (GC3) of codons as well as the average value of GC1 and GC2 (GC). The effective number of codons (ENC) was calculated using GHIPS. Relative synonymous codon usage (RSCU) was analyzed using CodonW1.4.4.

### 4.6. Gene Structure and Cis-Regulatory Element Analyses of PPO Gene Family Members

Based on the reported CDS sequence, the full-length gene file was obtained using activestate (https://www.activestate.com/ (accessed on 28 September 2022)) and GSDS (http://gsds.cbi.pku.edu.cn/ (accessed on 28 September 2022)). A structure map of the PPO family gene of *P. fortunei* was drawn.

Sequence information for the region 2000 bp upstream of *PfPPO* genes was extracted. The PlantCARE database (http://bioinformatics.psb.ugent.be/webtools/plantc-are/html/ (accessed on 11 October 2022)) was used to predict the roles of cisregulatory elements and TBtools was used to visualize the results.

### 4.7. Phylogenetic and Collinearity Analyses of the PPO Gene Family

A phylogeny of the PPO gene family in *P. fortune*, *Populus trichocarpa*, *Oryza sativa*, *Capsicum annuum*, *Glycine max*, *Triticum aestivum*, and *Zea mays* was reconstructed with the neighbor-joining method using MEGA-X 10.2 (Method, NJ; Bootstrap, 1000). And, the evolutionary tree of the PPO proteins was modified using the iTOL (https://itol.embl.de/ (accessed on 20 August 2022)) online website.

The local BLAST program was used to compare the protein sequences of PPO gene family members, and collinearity was evaluated (Evalue < −10). Use the online software MCScanX program to obtain the collinear file, and use the circle_plotter tool to visualize the result.

### 4.8. Analysis of Gene Expression in PPO Family Members of P. fortunei

Total RNA was extracted from the roots, stems, leaves, and top buds of PF, PFI seedlings, and leaves of PFT using a plant RNA extraction kit (Tiangen Biotech Co., Beijing, China). PfActin was used as the internal control. The reaction system and procedure followed those described by Cao et al. [56]. A real-time quantitative polymerase chain reaction (qRT-PCR) was used to evaluate *PfPPO* gene expression in different tissue parts of 10 healthy *P. fortunei* seedlings (Table 4). Relative gene expression was evaluated using the 2^−ΔΔCT^ method, with three biological replicates per sample. GraphPad Prism was used for analyses, and Primer-BLAST was used for primer design (https://www.ncbi.nlm.nih.gov/tools/primer-blast/ (accessed on 22 August 2022)).

### 4.9. Statistical Analysis

All results were collected from three parallel experiments. Data were compared with the control group or between treatments, by using the analysis ofvariance (ANOVA) and Duncan’s multiple range test with significant differences; for the Student’s *t*-test, * *p*< 0.05. Graphs were plotted using GraphPad Prism 8.0.

## 5. Conclusions

In summary, the *P. fortunei* genome includes 10 members of the *PfPPO* gene family. Similar to the gene family in other plants, *PfPPO* gene family members participate in multiple biological processes in *P. fortunei*. A transcriptome analysis showed that PPO gene expression is tissue-specific, and the same gene may have different functions in response to biotic and abiotic stresses. Specific gene functions need to be verified using transgenic plants. These results provide an important basis for breeding new varieties resistant to biotic and abiotic stresses.

## Figures and Tables

**Table 1:** Physical and chemical property analysis of *PfPPOs*.

Gene Name	Gene ID	Amino Acid	Relative Molecular Weight (kDa)	Isoelectric Point	Average Value of Total Hydrophilicity	Subcellular Localization	Chloroplast Transit Peptide
*PfPPO1*	Pfo09g005470	587	66.001	7.65	−0.532	chloroplast	√
*PfPPO2*	Pfo09g018160	592	66.587	6.10	−0.500	chloroplast	√
*PfPPO3*	Pfo09g018170	592	66.610	6.42	−0.525	chloroplast	√
*PfPPO4*	Pfo09g018180	592	66.649	6.02	−0.530	chloroplast	√
*PfPPO5*	Pfo09g018200	574	64.525	7.22	−0.533	peroxisome	-
*PfPPO6*	Pfo09g018210	576	64.097	5.68	−0.389	chloroplast	√
*PfPPO7*	Pfo10g006300	589	66.272	6.15	−0.358	chloroplast	-
*PfPPO8*	Pfo16g004540	593	66.677	6.72	−0.508	chloroplast	√
*PfPPO9*	Pfo16g004550	499	56.695	5.76	−0.423	cytoplasm	√
*PfPPO10*	Pfo16g004560	406	44.959	9.15	−0.357	chloroplast	√

Note: “√” indicates the presence of chloroplast transport peptides in the gene, and “-” indicates the absence of chloroplast transport peptides in the gene.

**Table 2:** Correlation parameter of codon usage bias in *PfPPOs*.

Gene	GC				ENC
	GC1	GC2	GC3s	GC	
*PfPPO1*	50.68	42.01	49.10	47.85	57.65
*PfPPO2*	51.26	39.63	53.90	48.79	56.87
*PfPPO3*	51.43	39.97	54.20	49.02	58.57
*PfPPO4*	51.60	39.97	52.70	48.62	57.88
*PfPPO5*	50.96	39.65	57.00	49.74	53.25
*PfPPO6*	54.59	41.94	62.00	53.26	53.49
*PfPPO7*	48.64	42.88	43.50	45.59	54.85
*PfPPO8*	52.19	40.40	58.30	50.84	57.01
*PfPPO9*	53.60	36.00	49.50	46.87	55.97
*PfPPO10*	50.37	48.65	54.80	52.01	57.94
Average value	51.53	41.11	53.50	49.26	56.35

Note: GC1, GC2, and GC3, respectively, represent the GC content of the first, second, and third bits of the codon; total GC represents the total GC content of the codon; ENC indicates the number of effective codons.

**Table 3:** Statistics of codon bias of *P. fortunei* PPO family genes.

Amino Acid	Codon	Number	Relative Synonymous Codon Usage	Amino Acid	Codon	Number	Relative Synonymous Codon Usage
Phe	UUU	103	0.80	Ser	**UCU**	82	1.32
	**UUC**	154	1.20		**UCC**	87	1.40
Leu	UUA	22	0.29		**UCA**	67	1.08
	**UUG**	139	1.80		UCG	54	0.87
	**CUU**	85	1.10		AGU	29	0.47
	CUC	72	0.93		AGC	54	0.87
	CUA	29	0.38	Pro	CCU	93	0.88
	**CUG**	116	1.50		**CCC**	116	1.09
Val	**GUU**	96	1.14		**CCA**	138	1.30
	GUC	78	0.93		CCG	77	0.73
	GUA	24	0.28	Thr	**ACU**	96	1.09
	**GUG**	139	1.65		**ACC**	113	1.28
Tyr	UAU	96	0.97		ACA	86	0.98
	**UAC**	102	1.03		ACG	57	0.65
His	CAU	66	0.98	Ala	**GCU**	117	1.23
	**CAC**	69	1.02		**GCC**	125	1.31
Gln	**CAA**	91	1.01		GCA	74	0.78
	CAG	90	0.99		GCG	65	0.68
Asn	AAU	128	0.86	Arg	CGU	41	0.92
	**AAC**	169	1.14		**CGC**	45	1.01
Lys	AAA	180	0.95		CGA	23	0.51
	**AAG**	199	1.05		CGG	25	0.56
Asp	GAU	206	0.99		**AGA**	63	1.41
	**GAC**	210	1.01		**AGG**	71	1.59
Glu	GAA	127	0.96	Ile	**AUU**	103	1.17
	**GAG**	137	1.04		**AUC**	102	1.15
Gly	GGU	76	0.96		AUA	60	0.68
	**GGC**	96	1.21	Met	AUG	117	1.00
	**GGA**	84	1.06	TER *	UAA	2	0.60
	GGG	61	0.77		UAG	2	0.60
Cys	UGU	25	0.55		**UGA**	6	1.80
	**UGC**	66	1.45	Trp	UGG	85	1.00

Note: * represents the stop codon; bold font represents RSCU > 1.

**Table 4:** Primer sequences required for the verification of differential genes of *PfPPOs’* family.

Gene Name	Forward Primer (5′–3′)	Reverse Primer (5′–3′)
*PfPPO1*	ATGACAGGCTTCGTGACCAG	CCGAAGAAAAGCCGAGGAGT
*PfPPO2*	TACAGTCACGACAATGCGCT	GCGCAGTGGACATTAGCTTG
*PfPPO3*	ACTAGACGTGAACTGCTGCC	ACTAGACGTGAACTGCTGCC
*PfPPO4*	TCTACGATGAGAACGCGCTG	CCTTCGGCCTGTTTGTCAAC
*PfPPO5*	TTGTGCTTACTGCAATGGCG	AAAACGGCAACGCGAAAGTT
*PfPPO6*	GGAAGGTCGACAGGAGGAAC	CTTGTCGAGCTCAGGTGGTT
*PfPPO7*	GACTCTGGTGATGACAGCCC	TGCTGCCGAAACCAATCAGA
*PfPPO8*	TGGGATTGACTGAGCTGCTG	ACCGCCAATGGTGATGTCTT
*PfPPO9*	GCTCGTGCGTGTTAAGGTTG	TGGAGCATTAGAAGTGGCGG
*PfPPO10*	CTTGAACACTGGCCAACAGC	ATCAGCAGGAAGGCGTTTCA
*PfActin*	AATGGAATCTGCTGGAAT	ACTGAGGACAATGTTACC

## Data Availability

Data are contained within the article.
